# Epicardial adipose tissue, cardiac damage, and mortality in patients undergoing TAVR for aortic stenosis

**DOI:** 10.1007/s10554-024-03307-4

**Published:** 2025-01-18

**Authors:** Pamela Piña, Daniel Lorenzatti, Annalisa Filtz, Andrea Scotti, Elena Virosta Gil, Juan Duarte Torres, Cristina Morante Perea, Leslee J. Shaw, Carl J. Lavie, Daniel S. Berman, Gianluca Iacobellis, Piotr J. Slomka, Philippe Pibarot, Marc R. Dweck, Damini Dey, Mario J. Garcia, Azeem Latib, Leandro Slipczuk

**Affiliations:** 1 Department of Cardiology, CEDIMAT, Santo Domingo, Dominican Republic; 2 Cardiology Division, Montefiore Medical Center, Albert Einstein College of Medicine, 111 E 210st, Bronx, NY USA; 3 Department of Cardiology, Araba-Txagorritxo University Hospital, Vitoria-Gasteiz, Spain; 4 Department of Cardiology, Gomez Ulla Central de la Defensa Hospital, Madrid, Spain; 5 Department of Cardiology, University Hospital of Toledo, Toledo, Spain; 6 Icahn School of Medicine at Mount Sinai, New York, NY USA; 7 Ochsner Clinical School, John Ochsner Heart and Vascular Institute, University of Queensland School of Medicine, New Orleans, LA USA; 8 Department of Imaging, Medicine, and Biomedical Sciences, Cedars-Sinai Medical Center, Los Angeles, CA USA; 9 Division of Endocrinology, Diabetes and Metabolism, Department of Medicine, University of Miami, Miami, FL USA; 10 Québec Heart and Lung Institute, Université Laval, Québec City, Québec Canada; 11 British Heart Foundation Centre for Cardiovascular Science, Edinburgh Heart Centre, University of Edinburgh, Edinburgh, UK

**Keywords:** CCTA, TAVR, TAVI, Epicardial adipose tissue, Aortic stenosis, Cardiac damage

## Abstract

Computed tomography (CT)-derived Epicardial Adipose Tissue (EAT) is linked to cardiovascular disease outcomes. However, its role in patients undergoing Transcatheter Aortic Valve Replacement (TAVR) and the interplay with aortic stenosis (AS) cardiac damage (CD) remains unexplored. We aim to investigate the relationship between EAT characteristics, AS CD, and all-cause mortality. We retrospectively included consecutive patients who underwent CT-TAVR followed by TAVR. EAT volume and density were estimated using a deep-learning platform and CD was assessed using echocardiography. Patients were classified according to low/high EAT volume and density. All-cause mortality at 4 years was compared using Kaplan-Meier and Cox regression analyses. A total of 666 patients (median age 81 [74–86] years; 54% female) were included. After a median follow-up of 1.28 (IQR 0.53–2.57) years, 11.7% (*n* = 77) of patients died. The EAT volume (*p* = 0.017) decreased, and density increased (*p* < 0.001) with worsening AS CD. Patients with low EAT volume (< 49cm^3^) and high density (≥-86 HU) had higher all-cause mortality (log-rank *p* = 0.02 and *p* = 0.01, respectively), even when adjusted for age, sex, and clinical characteristics (HR 1.71, *p* = 0.02 and HR 1.73, *p* = 0.03, respectively). When CD was added to the model, low EAT volume (HR 1.67 *p* = 0.03) and CD stages 3 and 4 (HR 3.14, *p* = 0.03) remained associated with all-cause mortality. In patients with AS undergoing TAVR, CT-derived low EAT volume, and high density were independently associated with increased 4-year mortality and worse CD stage. Only EAT volume remained associated when adjusted for CD.

**Graphical Abstract:**

**Figure:**
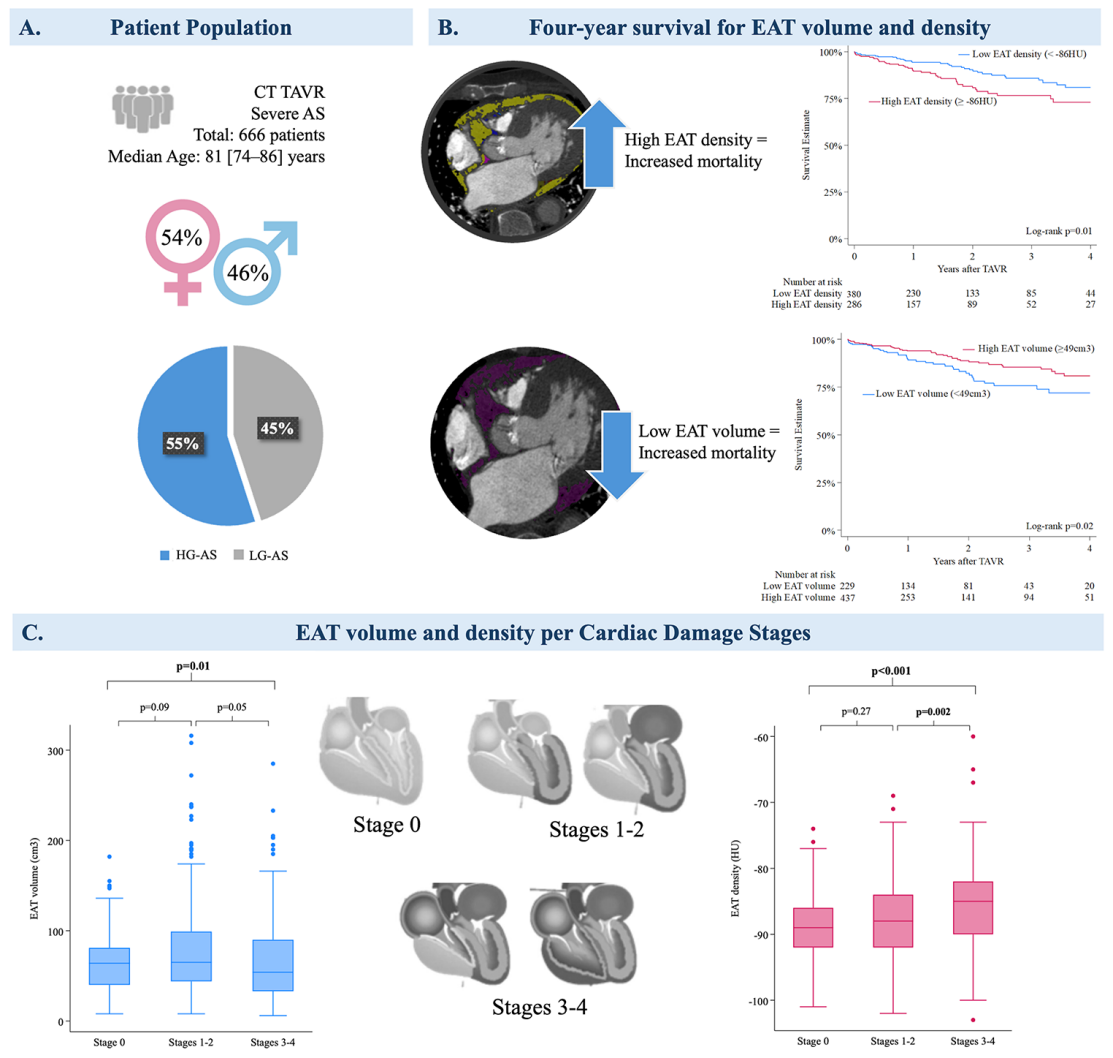
**Panel A** The patient population included 666 patients with symptomatic severe AS referred for computed tomography for TAVR planning. The median age of the cohort was 81 (IQR 74–86) years, with 54% being female. **Panel B**, Kaplan-Meier graphs demonstrate four-year survival for EAT volume and density with estimated cutoff values. **Panel C**, Box plots reveal median values and confidence intervals for EAT volume and density per cardiac damage stages. AS, aortic stenosis; EAT, epicardial adipose tissue; HU, Hounsfield units; EAT, epicardial adipose tissue

**Supplementary Information:**

The online version contains supplementary material available at 10.1007/s10554-024-03307-4.

## Introduction

Transcatheter aortic valve replacement (TAVR) has become an established therapy for severe aortic valve (AV) stenosis (AS) [1, 2]. Various clinical, echocardiographic [3, 4], and computed tomography (CT) factors [5, 6] have been identified as predictors of adverse outcomes following TAVR. Assessment of the extent of extravalvular cardiac damage (CD) by echocardiography has proven to be one of the strongest independent prognostic predictors of 1 and 5-year all-cause mortality [4, 7]. However, this has not been properly tested in truly diverse populations.

Effective planning using cardiac CT imaging is essential for achieving optimal outcomes both during the procedure and in the early post-procedural phase. However, data for the prediction of long-term outcomes are scarce [8]. Advanced imaging markers, such as epicardial adipose tissue (EAT) have garnered significant attention as a risk predictor of cardiovascular disease (CVD) [9] and all-cause mortality in TAVR patients [10]. This unique fat depot has paracrine and vasocrine interactions with the heart and has been shown to play a role in the development of CVD, such as coronary artery disease, atrial fibrillation, and heart failure (HF). Importantly, EAT has a dynamic nature as it is subject to variations related to several factors such as age, race/ethnicity background [11, 12], certain pathological settings, and response to medical therapy [13–15]. Particularly in patients with progressive calcific AS, EAT could also act as a source of detrimental inflammatory mediators [16, 17].

Computed tomography stands out among imaging methods for quantifying EAT due to its high spatial resolution and complete cardiac coverage. It can also provide insightful data on EAT density, which has recently emerged as a marker of inflammation and an independent predictor of atherosclerosis [18]. Furthermore, almost every patient considered for TAVR therapy undergoes CT imaging, representing an opportunity to study the interplay between the amount and quality of EAT and post-procedural long-term outcomes in this population. However, to date, only a few studies have looked at the relationship between EAT and long-term outcomes in this population [10, 19–21]. Furthermore, data on diverse populations and the link between EAT and CD are lacking.

This study aimed to investigate the interaction of EAT volume and density with CD and mortality in patients who underwent TAVR for severe AS.

## Methods

The study protocol followed the ethical guidelines of the 1975 Declaration of Helsinki and was approved in advance by our Institutional Review Board (Office of Human Research Affairs) at Albert Einstein College of Medicine. Given the study design, the requirement for informed consent was waived. Data is available upon reasonable request, subject to institutional approval.

### Patient population

Patients were retrospectively identified from our Cardiovascular CT registry. All consecutive patients undergoing CT-TAVR between February 2015 and June 2022 at Montefiore Health Network (New York, USA) were included. Patients were excluded for the following reasons: poor-quality images (significant breathing motion, metal artifacts from sternal wires or intracardiac devices), prior surgical aortic valve replacement, and TAVR not performed. Baseline and outcome data were collected through chart review.

Race/ethnicity was self-identified at the time of registration in medical records. Ethnicity was categorized as Hispanic or non-Hispanic. Within the non-Hispanic group, race was classified as non-Hispanic White, non-Hispanic Black, non-Hispanic Asian, or other/unknown.

### CT image acquisition

Patients underwent standardized contrast-enhanced CT for pre-procedural aortic annulus sizing and vascular access assessment as recommended by current expert consensus [22]. Images were acquired at three different scanners (64-slice GE Lightspeed VCT, Optima CT660, and 64-slice Philips IQon) using 70 to 155 mL of Isovue-370 contrast. All studies were performed using 120 kVp as per the institutional protocol, and the tube current (mA) was adjusted according to the patient’s body mass index (BMI) as per scanner protocol.

### Measurement of EAT

Measurements were performed on the retrospective ECG-gated axial reconstructions at 75% of the R-R interval with slice thickness/increment of 0.625/0.625 mm and using a standard convolution kernel. For quantification of EAT volume, images in DICOM format were exported to a stand-alone workstation with dedicated software (QFAT; v2.0; Cedars-Sinai Medical Center, Los Angeles, CA, USA) [23, 24]. QFAT uses convolutional deep-learning for fully automated quantification of EAT volume (Figure S1). The pericardium was traced semi-automatically from the level of the bifurcation of the pulmonary trunk to that of the diaphragm. Thresholds (− 195 to − 15 HU) were used to define fat voxels within the epicardium as previously suggested for EAT volume measurements on CT angiography scans [25]. EAT was measured as the fat depot between the myocardium and visceral layer of the pericardium to differentiate from pericardial fat. Manual adjustments were performed when necessary, and total volumes and mean density (attenuation) of EAT in cubic centimeters and Hounsfield Units (HU) were documented.

### Data collection

Patients were assigned to distinct groups of AS (e.g., low-gradient [LG-AS] or high-gradient [HG-AS]) following guideline recommendations [26]. High-gradient AS was defined as maximum velocity (Vmax) ≥ 4 m/s and/or mean pressure gradient (PG) ≥ 40 mmHg with aortic valve area (AVA) ≤ 1 cm^2^, whereas LG-AS was defined as Vmax < 4 m/s and/or mean PG < 40 mmHg with AVA ≤ 1cm^2^ regardless of LV ejection fraction (LVEF). Clinical parameters, including CD stage, CVD risk factors, prior medical history, and laboratory markers, were retrospectively collected from the CT Scan encounter and closest clinic visit. The last systematic follow-up assessment was performed in November 2022 using electronic medical records.

### Cardiac damage stages

Patients were categorized into five stages (independent, not additive) depending on the presence or absence of extra-valvular (extra-aortic valve) CD or dysfunction using transthoracic echocardiography as previously described [4]. Stages were defined as follows: stage 0: No other CD detected; stage 1: LV damage as defined by the presence of LV hypertrophy, severe LV diastolic dysfunction, or LV systolic dysfunction (LVEF < 50%); stage 2: LA or mitral valve damage or dysfunction; stage 3: Pulmonary artery vasculature or tricuspid valve damage or dysfunction and stage 4: RV damage as defined by the presence of moderate or severe RV dysfunction.

### Statistical analysis

Data are presented as median and interquartile range (IQR) for non-normally distributed continuous variables. Categorical variables are presented as counts and percentages. Continuous variables with a non-parametric distribution were compared using the Mann-Whitney-U test.

The primary outcome was all-cause mortality four years after TAVR. We performed a Kaplan-Meier survival analysis with median values for EAT volume and density, EAT volume indexed by height, and with a binary cut-off estimated using the Youden index for both EAT volume and density. The log-rank p-value was used to assess differences between groups. Univariable Cox regression analysis was performed to obtain hazard ratios (HR) with 95% confidence intervals for cumulative mortality rates four years after TAVR. Multivariable models were built incrementally using covariates that were considered clinically relevant to patients with AS. The first model included age, sex, and BMI, then cardiovascular risk factors were added, and finally, a third model was built, including CD stages, for EAT volume and density. The relationship between EAT and mortality was further explored using Cox regression analysis by entering the EAT volume measurement as a restricted cubic spline with 3 knots. Statistical significance was set at *p* < 0.05. Statistical analyses were performed using Stata 17 software (StataCorp, College Station, TX).

## Results

### Study population

Overall, 666 patients with available pre-intervention CT-TAVR images who underwent TAVR were included in this analysis. During follow-up (median 1.28 [IQR 0.53–2.57] years), 77 patients died (11.7%). The median age of the cohort was 81 (74–86) years, and 54% of the patients were female. Our cohort included racially/ethnically diverse patients, with 40% Non-Hispanic White, 24% Hispanic, and 13% Non-Hispanic Black. The median EAT volume was 63 (40–92) cm^3^, and the median EAT density was − 87 (-92-[-83]) HU. Comorbidities, CVD risk factors, and echocardiographic characteristics according to race/ethnicity are described in Table 1 and divided by sex in Table S1.

**Table 1:** Baseline demographics stratified per race/ethnicity

	Total (*n* = 666)	Non-hispanic black (*n* = 88)	Hispanic (*n* = 158)	Non-hispanic white (*n* = 265)	Other/Unk (*n* = 155)	*p*-value
Age	81 (74–86)	80 (72–84)	78 (70–83)	82 (77–87)	81 (74–87)	**< 0.001**
Sex						**< 0.001**
Female	357 (54%)	67 (76%)	74 (47%)	134 (51%)	82 (53%)	
BMI (kg/m^2^)	28 (24–32)	29 (26–35)	28 (24–32)	27 (24–31)	27 (23–32)	**0.005**
BMI Categories						0.085
0 (< 25 kg/m^2^)	213 (32%)	18 (20%)	48 (30%)	95 (36%)	52 (34%)	
1 (25–30 kg/m^2^)	230 (35%)	30 (34%)	56 (35%)	94 (35%)	50 (32%)	
2 (≥ 30 kg/m^2^)	223 (33%)	40 (45%)	54 (34%)	76 (29%)	53 (34%)	
HTN	320 (48%)	58 (66%)	92 (58%)	120 (45%)	50 (32%)	**< 0.001**
Diabetes	151 (23%)	33 (38%)	52 (33%)	42 (16%)	24 (15%)	**< 0.001**
Dyslipidemia	266 (40%)	52 (59%)	73 (46%)	96 (36%)	45 (29%)	**< 0.001**
CAD	124 (19%)	15 (17%)	36 (23%)	52 (20%)	21 (14%)	0.19
ACS	10 (2%)	2 (2%)	3 (2%)	2 (1%)	3 (2%)	0.63
CKD	94 (14%)	22 (25%)	29 (18%)	21 (8%)	22 (14%)	**< 0.001**
Stroke	37 (6%)	5 (6%)	14 (9%)	12 (5%)	6 (4%)	0.20
EF %	60 (55–65)	60 (50–65)	64 (55–65)	60 (55–65)	60 (55–65)	0.62
AVA (continuity, cm^2^)	0.73 (0.60–0.85)	0.74 (0.59–0.86)	0.74 (0.62–0.86)	0.71 (0.61–0.84)	0.73 (0.60–0.85)	0.75
Ao mean PG (mmHg)	39 (31–46)	39 (32–46)	38 (31–47)	39 (31–46)	40 (31–47)	0.88
AS max vel (cm/s)	400 (360–440)	399 (370–428)	398 (346–441)	400 (360–442)	403 (361–442)	0.97
Valve morphology						0.35
Bicuspid	37 (6%)	3 (3%)	13 (8%)	14 (5%)	7 (5%)	
AS Severity						0.40
LG-AS	303 (45%)	41 (47%)	80 (51%)	118 (45%)	64 (41%)	
HG-AS	363 (55%)	47 (53%)	78 (49%)	147 (55%)	91 (59%)	
Cardiac Damage Stage						0.29
0	93 (14%)	11 (12%)	21 (13%)	40 (15%)	21 (14%)	
1	46 (7%)	5 (6%)	9 (6%)	20 (8%)	12 (8%)	
2	312 (47%)	34 (39%)	78 (49%)	126 (48%)	74 (48%)	
3	180 (27%)	27 (31%)	45 (28%)	67 (25%)	41 (26%)	
4	35 (5%)	11 (12%)	5 (3%)	12 (5%)	7 (5%)	
EAT volume (cm^3^)	63 (40–92)	41 (29–62)	64 (45–92)	69 (44–101)	61 (40–97)	**< 0.001**
EAT density (HU)	-87 (-92–83)	-87 (-91–83)	-88 (-92–83)	-87 (-92–84)	-88 (-92–83)	0.76

AS, aortic stenosis; ACS, acute coronary syndrome; AVA, aortic valve area; BMI, body mass index; CAD, coronary artery disease; CKD, chronic kidney disease; EAT, epicardial adipose tissue; EF, ejection fraction; HG, high-gradient; HTN, hypertension; HU, Hounsfield units; LG, low-gradient; PG, pressure gradient; Unk, Unknown

### Echocardiographic characteristics

In the overall cohort, the median AV peak velocity was 400 cm/s (360–440 cm/s) with a median mean gradient of 39 mmHg (31–46 mmHg) and a median estimated AVA of 0.73 cm^2^ (0.60–0.85 cm^2^). Most (84%) had LVEF ≥ 50%. Regarding CD, only 14% had no echocardiographic evidence of extravalvular damage, while most patients were in stages 2 (47%) and 3 (27%). The echocardiographic variables are presented in Tables 1and Table S2.

### EAT volume, AV hemodynamics, and cardiac damage

Using the optimal EAT volume binary cut-off as determined by Youden index (Figure S2), patients were dichotomized into high and low EAT volume groups (≥ 49 cm^3^ vs. <49 cm^3^). Those in the low EAT volume group were more commonly female and non-Hispanic Black (*p* < 0.001)(Table S3). Additionally, they had lower BMI (25 vs. 29 kg/m^2^; *p* < 0.001) and were more commonly in CD stage 3 (*p* = 0.01) (Figure S3). Comorbidities and CVD risk factors were equally distributed between both groups, as were AS hemodynamic severity and LVEF (Figure S4).

Table S4 summarizes the baseline characteristics and echocardiographic indices according to AS type. There were no differences in the EAT volume, between the AS hemodynamic phenotype groups (EAT volume = 62cm^3^ in LG-AS vs. 63 cm^3^ in HG-AS; *p* = 0.87). The EAT volume decreased significantly (*p* = 0.017), as did the indexed volume (*p* = 0.013), in more advanced stages of CD (Figure S5). History of chronic kidney disease (CKD) and diabetes was more prevalent in those patients with stages 3 and 4 (Table S5).

### EAT density, AV hemodynamics, and cardiac damage

Patients were dichotomized into high and low EAT density groups using the optimal EAT density binary cut-off determined by the Youden index (≥ -86 HU vs. < -86 HU). Patients in the high EAT density group were significantly older (*p* = 0.002), had a lower BMI (*p* < 0.001), had a prior history of CKD (*p* = 0.01) and stroke (*p* = 0.03), and were classified more frequently into CD stages 3 (*p* < 0.001) and 4 (*p* = 0.03) than those in the low EAT density group (Table S6). Moreover, EAT density increased with advancing stages of CD (*p* < 0.001) (Figure S5).

### EAT and mortality

**Fig. 1:**
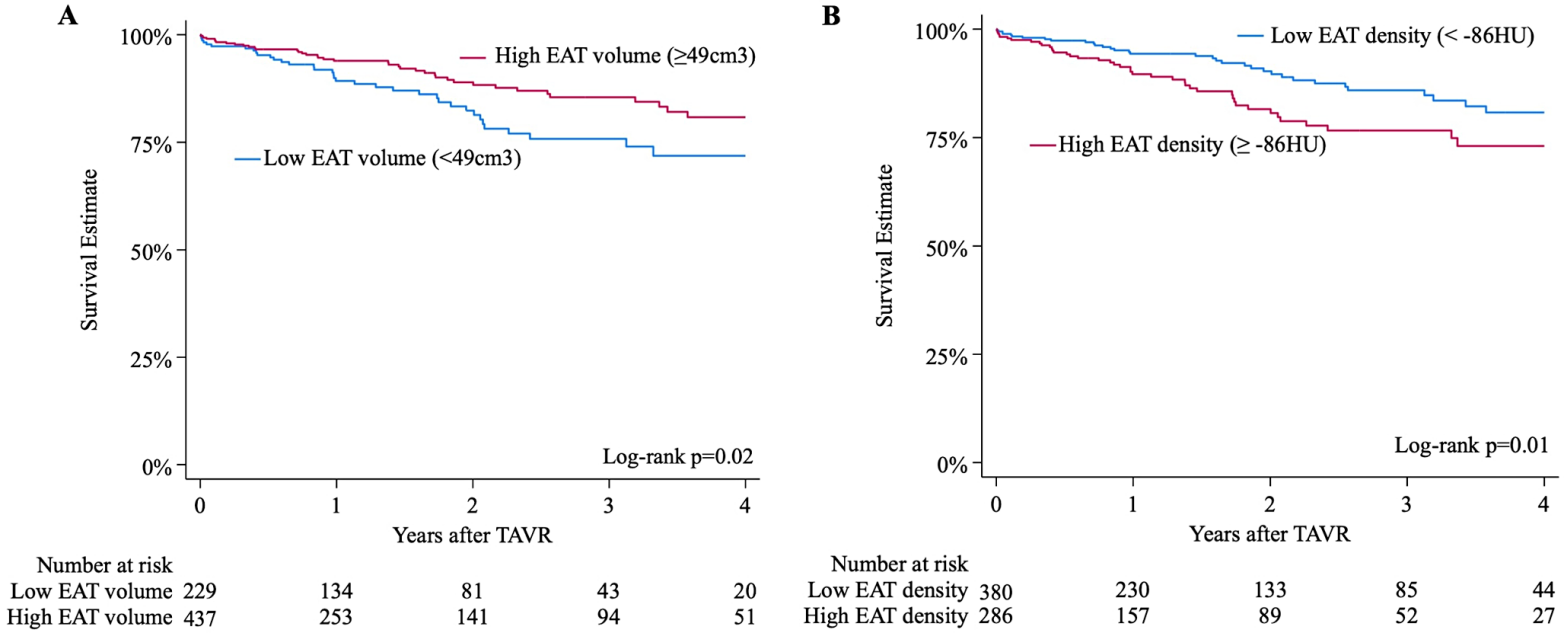
Kaplan-Meier graph demonstrating four-year survival for EAT volume (**A**) and density (**B**) with estimated cutoff values. EAT: epicardial adipose tissue, HU: Hounsfield units

**Fig. 2:**
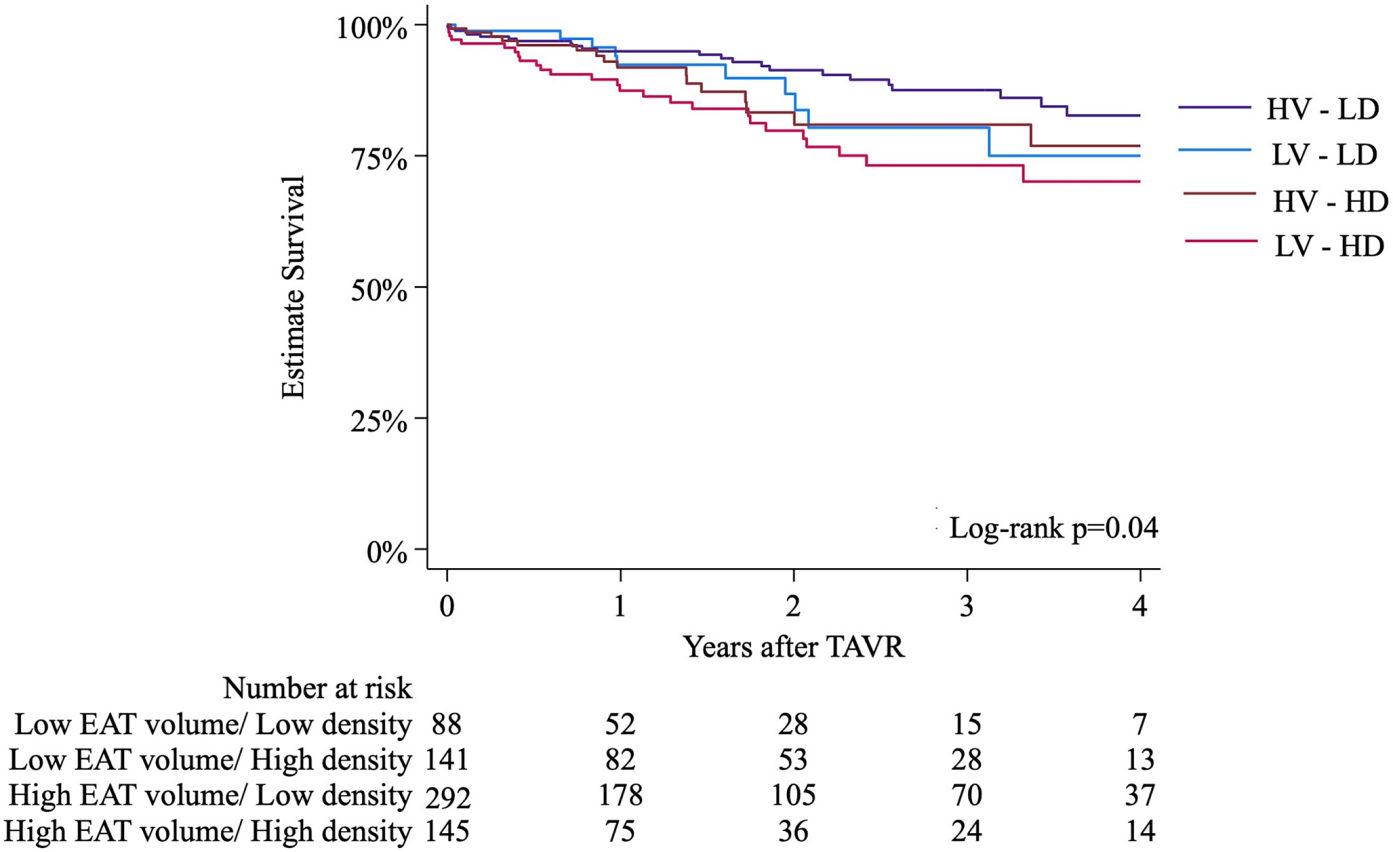
Kaplan-Meier graph demonstrating four-year survival for 4 subgroups according to combined EAT volume and density cut-offs. HD: high density, HV: high volume, LD: low density, LV: low volume

Patients with low EAT volume had significantly higher all-cause mortality than those with high EAT volume (log-rank *p* = 0.02) (Fig. 1A). Even when indexed by height, patients with low-indexed EAT volume (< 37 cm^3^/m) still had higher mortality than those with high-indexed EAT volumes (≥ 37 cm^3^/m), especially men compared to women (Figure S6). Similarly, higher EAT density was associated with increased all-cause mortality (log-rank *p* = 0.01) (Fig. 1B). When stratified by the possible subgroups of combined volume and density cut-offs, patients with simultaneous low-volume and high-density EAT had the highest mortality (log-rank *p* = 0.04)(Fig. 2).

In a multivariable Cox regression analysis, after adjustment for age, sex, and BMI, low EAT volume (HR 1.76, 95% CI 1.09–2.84, *p* = 0.02) and high EAT density (HR 1.73, 95% CI 1.08–2.77, *p* = 0.01) remained significantly associated with mortality (Table 2).

**Fig. 3:**
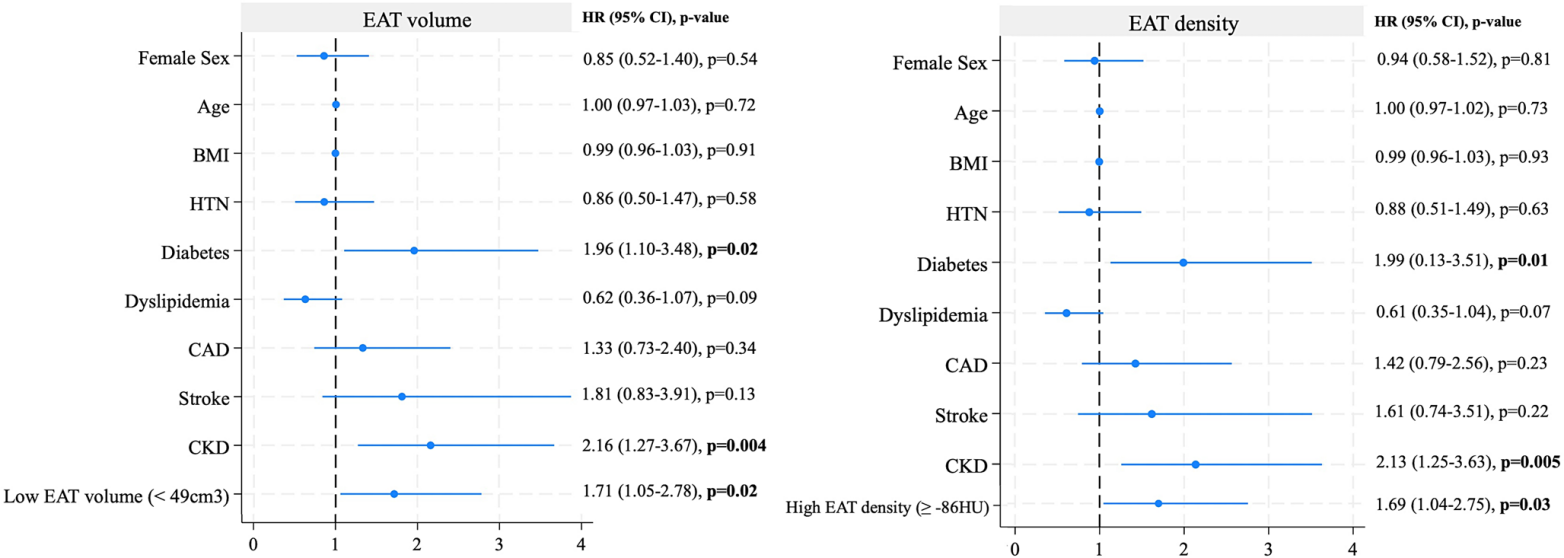
Multivariable Cox regression analysis in models including cardiovascular risk factors associated with mortality for EAT volume and density. BMI, body mass index; CAD, coronary artery disease; CKD, chronic kidney disease; EAT, epicardial adipose tissue; HTN, hypertension; HU, Hounsfield units

**Fig. 4:**
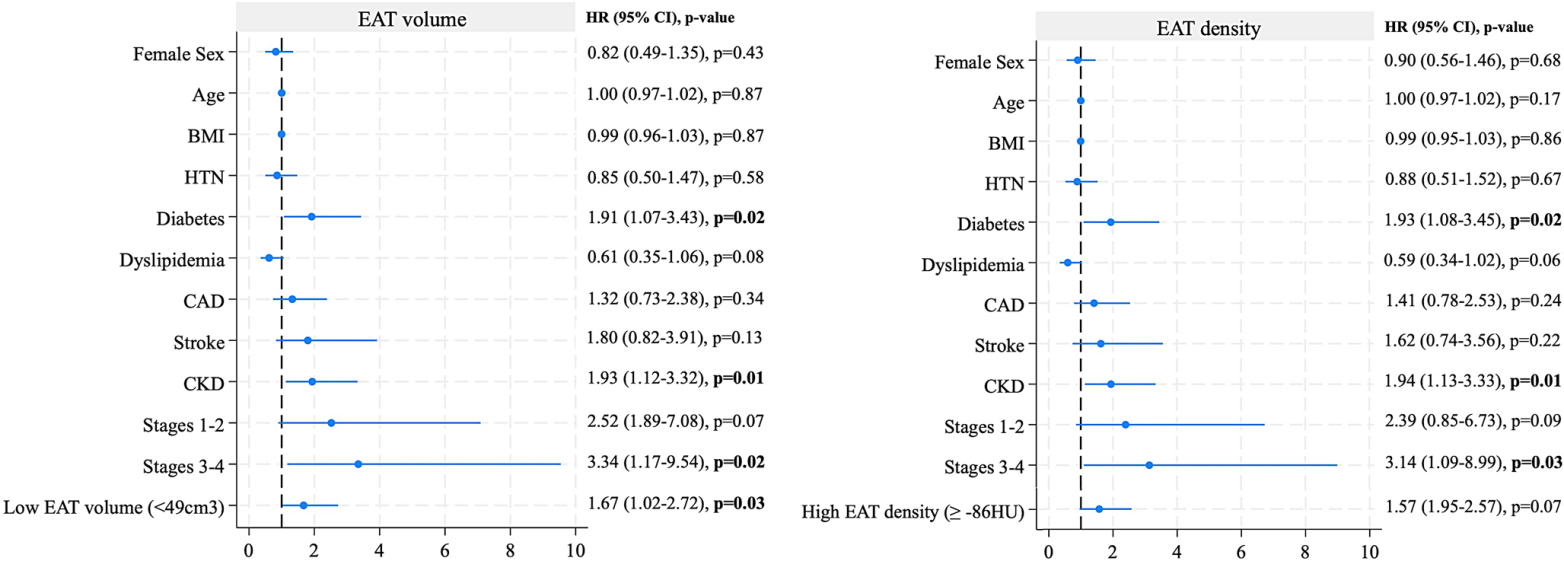
Multivariable Cox regression analysis in models including cardiovascular risk factors and cardiac damage associated with mortality for EAT volume and density. BMI, body mass index; CAD, coronary artery disease; CKD, chronic kidney disease; EAT, epicardial adipose tissue; HTN, hypertension; HU, Hounsfield units

In univariable Cox regression analysis, the presence of CKD (HR 2.41; *p* = 0.001) and CD stage 3 or 4 (HR 3.94; *p* = 0.01) were associated with increased mortality after TAVR (Table 3). When a second incremental model was built, including age, sex, BMI, and comorbidities, low EAT volume and high EAT density remained independently associated with all-cause mortality (HR 1.71, 95% CI [1.05–2.78]; HR 1.69 CI [1.04–2.75], respectively; Fig. 3). In the third incremental model, including all previously adjusted variables plus CD, EAT volume but no density remained independently associated with increased mortality(HR 1.67 CI [1.02–2.72], *p* = 0.03) (Fig. 4). Additionally, there was a stepwise increase in mortality with progressively worse CD stage (log-rank *p* = 0.01; Figure S7). Restricted cubic spline regression analysis showed a linear relationship between EAT volume, density, and mortality (nonlinearity *p* = 0.421 and *p* = 0.29, respectively; Figure S8).

**Table 2:** Uni- and multivariable Cox-regression models with EAT volume and density association with mortality

Variable	Univariable HR (95% CI)	*p*-value	Multivariable HR* (95% CI)	*p*-value
Low EAT volume (< 49cm^3^)	1.65 (1.05–2.59)	**0.02**	1.76 (1.09–2.84)	**0.02**
High EAT density (≥-86HU)	1.70 (1.09–2.67)	**0.01**	1.73 (1.08–2.77)	**0.01**

*Adjusted for age, sex, and body mass index. EAT, epicardial adipose tissue; HR, hazard ratio; HU, Hounsfield Units

**Table 3:** Univariable cox-regression model with cardiovascular risk factors and CD association with mortality

Variable	Univariable HR (95% CI)	*p*-value
Age	0.99 (0.97–1.01)	0.68
Female sex	0.85 (0.50–1.34)	0.50
BMI	0.98 (0.95–1.02)	0.55
Hypertension	1.05 (0.67–1.65)	0.81
Diabetes	1.57 (0.97–2.54)	0.06
Dyslipidemia	0.76 (0.47–1.21)	0.25
CAD	1.30 (0.75–2.23)	0.33
Stroke	1.93 (0.93–4.03)	0.07
CKD	2.41 (1.44–4.02)	**0.001**
Cardiac Damage Stages		
0	ref.	
Stages 1–2 Stages 3–4	2.57 (0.91–7.19) 3.94 (1.39–11.13)	0.07 **0.01**
EAT volume (< 49cm^3^)	1.65 (1.05–2.59)	**0.02**
EAT density (≥ -86HU)	1.70 (1.09–2.76)	**0.01**

The **bold** text indicates a significant p-value. CAD, coronary artery disease; CKD, chronic kidney disease; EAT, epicardial adipose tissue; HR, hazard ratio; HU, Hounsfield Units

## Discussion

Our study represents the largest investigation to date on the association of CT-derived EAT volume and density with postprocedural outcomes after TAVR. Moreover, it is the first of its kind in a US-based cohort comprising diverse race/ethnic backgrounds and with simultaneous cardiac damage assessment. The main findings of our study can be summarized as follows: (1) Low EAT volume (< 49 cm3) was independently associated with higher 4-year all-cause mortality, even when adjusted for CD, and contrary to what has been demonstrated in previous literature; (2) Higher EAT density (≥ -86 HU) was also associated with increased all-cause mortality, independent of clinical variables but not when AS cardiac damage stage was accounted; (3) EAT volume progressively decreased, and EAT density increased with worsening AS cardiac damage stage; and (4) AS cardiac damage stage was the strongest independent predictor of mortality in this diverse cohort.

In the widespread TAVR era, CT has become an indispensable tool for adequate patient selection and accurate preprocedural TAVR planning based on its ability to predict intraprocedural and early postprocedural complications [27–31]. In addition, CT could provide a more comprehensive phenotype involving not only the valve and myocardium but also more systemic disease markers such as the psoas muscle, subcutaneous fat, and bone density, all of which have shown a direct impact on longer-term outcomes [6, 32–34]. In this context, EAT could also serve as an opportunistic imaging biomarker for further refining TAVR therapy candidates or guiding patient postprocedural care.

The ultimate mechanistic explanation for EAT and its interplay with different CVD diseases remains a matter of debate [35, 36]. It is accepted that the nature of this relation is dynamic and varies with the age and severity of certain pathological conditions [9].

Our observation that lower (in contrast to higher) EAT volumes were associated with higher mortality represents a unique perspective to the existing literature [10, 20]. We believe this could be explained by the fact that we evaluated a population with more severe disease progression, as depicted by cardiac damage stage ≥ 2 in the vast majority of patients. In this specific setting, EAT may become dysfunctional and incur fibrotic and apoptotic changes, leading to an overall lower volume. Previous observational studies have found a significant reduction in both EAT volume and thickness among patients with HF with reduced LVEF (HFrEF) compared with both healthy individuals and those with preserved ejection fraction [35]. Furthermore, reduced EAT in HFrEF patients has been linked to worse LV function, adverse myocardial remodeling, and myocardial damage by troponins [37]. This observation supports the hypothesis that the reduction in EAT volume can be attributed to cardiac cachexia and catabolic-related adverse effects [35]. Large studies from North America, Europe, and Asia have shown that underweight patients undergoing TAVR exhibit higher rates of short and mid-term mortality than normal or overweight individuals, thereby supporting the hypothesis of a cardiovascular obesity paradox in this population [38–40]. However, our findings were independent of patient’s BMI and remained significant after adjusting for EAT volume by height.

Additionally, in patients with more advanced or end-stage organ disease and elderly individuals, the thermogenic function of EAT can be decreased, with reciprocal increases in the expression of genes encoding profibrotic and pro-apoptotic factors [9, 41]. The present study extended these observations to patients with severe AS undergoing TAVR.

Interestingly, higher EAT density, likely reflecting increased inflammatory activity, was strongly associated with mortality even after adjusting for age, sex, and BMI. This observation aligns with recent work by Salam et al. [19] in 1,197 patients undergoing TAVR. The authors found that a higher EAT density (>-81 HU) was strongly associated with increased 2-year mortality independent of clinical characteristics and surgical risk scores. In another recent study by Sato et al. [21] among 125 consecutive TAVR patients, authors showed higher EAT density (>-74.3 HU) predicted the occurrence of major adverse cerebral and cardiovascular events (MACCE) (AUC = 0.685) and the EAT density and EuroSCORE were independently associated with MACCE. Recently, EAT density was found to be related to COVID-19 severity, independent of the presence of coronary artery disease [42]. These findings are supported by the concept that aging, environmental factors, and genetics can lead to EAT adipocyte dysfunction, producing pro-inflammatory adipokines implicated in CVD pathogenesis [9]. However, the relationship between EAT density and CVD, especially for coronary atherosclerosis, has been considered controversial, with some studies showing direct and other inverse correlation [43].

In the context of aortic stenosis, EAT could contribute to the progression of valvular calcification and adverse ventricular remodeling through paracrine effects [44]. Parisi et al. [16]. enrolled 95 patients with severe calcific AS who underwent cardiac surgery for AVR. EAT thickness was assessed using echocardiography in these patients, and inflammatory profiles were analyzed using cytokines measurements. EAT thickness was significantly higher in patients with AS than in the control group. Notably, the EAT secretome of patients with increased EAT thickness showed higher levels of inflammatory mediators. Furthermore, the thickness of EAT significantly correlated with the levels of different pro-inflammatory and pro-atherogenic cytokines, such as IL-6, TNF-α, MCP-1, and IL-1β, therefore, the greater the thickness of EAT, the greater the secretion of these mediators. Interestingly, we found that patients with lower volume but more inflamed (higher density) EAT did worse than those with high volume and density, perhaps reflecting an advanced stage of the disease where there is persistent inflammation despite the decrease in the volume/thickness of the adipose tissue. Interestingly, a pro-inflammatory activation of EAT in patients with AS and EAT involvement in aortic valve calcific degeneration has been suggested [44].

While the correlation between greater extravalvular damage and increased mortality is not a novel finding, we believe that the specific composition of our cohort, including its diversity in terms of race/ethnicity and socioeconomic status, offers a unique perspective on interpreting this relationship. For instance, in the major TAVR trials, race/ethnicity was only reported in the PARTNER 3 trial, with non-White patients being only 7.7% in the TAVR and 9.9% in the surgical AVR group [45]. The reason for the underrepresentation of ethnic groups in trials is not clear, but we also know that in clinical practice, non-Hispanic Black and Hispanic patients are less likely to receive TAVR therapy, even within metropolitan areas [46].

The prognostic impact of EAT volume was independent of CD stage, but EAT density was not. This could be explained by the fact that cardiac damage remains one of the strongest independent prognostic markers in this population, establishing a high bar for any increased prognostic value [4, 47]. Additionally, EAT density quantification is less standardized than volume, as it is known to vary under the influence of CT acquisition parameters and the presence or absence of contrast [43]. Interestingly, the fact that EAT volume decreased and EAT density increased with worsening CD stage could be related to the role of EAT as a marker of more severe cardiac remodeling and the failure of the adaptation mechanisms to AS [48].

Since preprocedural CT imaging is routinely performed for TAVR planning, EAT quantification could provide incremental risk prediction without additional testing or radiation exposure. However, standardized EAT thresholds are still needed, given the overlap in values between patients with and without CVD events [10]. Additionally, the influence of contrast-enhanced images in EAT volume assessment should be considered. Variations of the lower threshold have been reported to have a negligible effect on the total EAT volume [49]. Bucher et al. reported significant differences in epicardial fat volume between non-contrast and contrast-enhanced (130.7 ± 49.5 ml vs. 87.2 ± 38.5 ml, *p* < 0.001) data sets at a − 30 HU upper threshold. Mean EAT volume for contrast-enhanced data sets at a -15 HU upper threshold (102.4 ± 43.6 ml) could be approximated most closely by non-contrast scans at a -45HU upper threshold (105.3 ± 40.8 ml) [25]. In our series, an upper threshold of -15 HU was used.

### Limitations

Our study is limited by the retrospective and observational nature of our findings, precluding causal inference and the presence of residual confounding that may have influenced our results despite the efforts made to account for those. The impact of medications, biomarkers, and/or frailty scale on clinical outcomes is unknown. In addition, since only patients with severe AS undergoing TAVR were studied, it remains unclear whether findings apply to those with surgical aortic valve replacement, conservative treatment, or those who died before receiving any treatment. Finally, we selected all-cause mortality as our primary outcome to improve statistical power and minimize potential biases in determining specific causes of death. This context is crucial for interpreting our findings accurately.

Prospective studies with larger cohorts and longer follow-up periods are needed to validate our findings and elucidate the underlying mechanisms driving the observed associations.

## Conclusion

In conclusion, our study demonstrated that in patients with severe AS undergoing TAVR, lower EAT volumes, and higher EAT density were both independently associated with increased postprocedural mortality and more severe preprocedural AS CD stages, which also remained strongly related to mortality. The potential value of these imaging markers lies in the fact that they can be rapidly and easily obtained from universally performed pre-TAVR CT. Further prospective studies are required to validate these findings.

## Electronic Supplementary Material

Below is the link to the electronic supplementary material.

Supplementary Material 1

## Data Availability

No datasets were generated or analysed during the current study.

## Abbreviations

AS: Aortic stenosis
AVA: Aortic valve area
CT: Computed tomography
EAT: Epicardial adipose tissue
HG-AS: High-gradient aortic stenosis
HFrEF: Heart failure with reduced ejection fraction
LG-AS: Low-gradient aortic stenosis
TAVR: Transcatheter aortic valve replacement
