# Supplementary material for: Epicardial adipose tissue, cardiac damage, and mortality in patients undergoing TAVR for aortic stenosis
Source: Int J Cardiovasc Imaging. 2025 Jan 18;41(2):279–90. doi: 10.1007/s10554-024-03307-4 (PMC11811257; doi:10.1007/s10554-024-03307-4)
Supplement: Supplementary file 1 — Supplementary Material 1 [file 10554_2024_3307_MOESM1_ESM.docx]

SUPPLEMENTAL MATERIAL

| **Supplemental Table 1. Baseline demographics per sex** | | | |  |
| --- | --- | --- | --- | --- |
|  | **Total (n=666)** | **Female (n=357)** | **Male (n=309)** | **p-value** |
| Age | 81 (74-86) | 81 (74-86) | 80 (74-86) | 0.29 |
| Race/Ethnicity |  |  |  | **<0.001** |
| Non-Hispanic  Black | 88 (13%) | 67 (19%) | 21 (7%) |  |
| Hispanic | 158 (24%) | 74 (21%) | 84 (27%) |  |
| Other/Unknown | 155 (23%) | 82 (23%) | 73 (24%) |  |
| Non-Hispanic White | 265 (40%) | 134 (38%) | 131 (42%) |  |
| BMI (kg/m^2^) | 28 (24-32) | 29 (24-34) | 27 (24-30) | **<0.001** |
| BMI Categories |  |  |  | **0.002** |
| 0 (<25 kg/m^2^) | 213 (32%) | 104 (29%) | 109 (35%) |  |
| 1 (25-30 kg/m^2^) | 230 (35%) | 112 (31%) | 118 (38%) |  |
| 2 (≥30 kg/m^2^) | 223 (33%) | 141 (39%) | 82 (27%) |  |
| HTN | 320 (48%) | 169 (47%) | 151 (49%) | 0.69 |
| Diabetes | 151 (23%) | 85 (24%) | 66 (21%) | 0.45 |
| Dyslipidemia | 266 (40%) | 144 (40%) | 122 (39%) | 0.82 |
| CAD | 124 (19%) | 41 (11%) | 83 (27%) | **<0.001** |
| ACS | 10 (2%) | 5 (1%) | 5 (2%) | 0.82 |
| CKD | 94 (14%) | 45 (13%) | 49 (16%) | 0.23 |
| Stroke | 37 (6%) | 18 (5%) | 19 (6%) | 0.53 |
| EF % | 60 (55-65) | 65 (59-65) | 60 (50-65) | **<0.001** |
| AVA (continuity, cm^2^) | 0.73 (0.60 -0.85) | 0.70 (0.57-0.82) | 0.76 (0.64-0.86) | **<0.001** |
| Ao mean PG (mmHg) | 39 (31-46) | 40 (32-48) | 38 (30-45) | **0.028** |
| AS max vel (cm/s) | 400 (360-440) | 400 (363-443) | 398 (358-440) | 0.33 |
| Valve morphology |  |  |  | **0.020** |
| Bicuspid | 37 (6%) | 13 (4%) | 24 (8%) |  |
| AS severity |  |  |  | **0.036** |
| LG-AS | 303 (45%) | 149 (42%) | 154 (50%) |  |
| HG-AS | 363 (55%) | 208 (58%) | 155 (50%) |  |
| Cardiac Damage  Stage |  |  |  | 0.38 |
| 0 | 93 (14%) | 44 (12%) | 49 (16%) | 0.19 |
| 1 | 46 (7%) | 21 (6%) | 25 (8%) | 0.26 |
| 2 | 312 (47%) | 170 (48%) | 142 (46%) | 0.67 |
| 3 | 180 (27%) | 100 (28%) | 80 (26%) | 0.54 |
| 4 | 35 (5%) | 22 (6%) | 13 (4%) | 0.26 |
| EAT volume (cm^3^) | 63 (40-92) | 52 (33-77) | 73 (49-110) | **<0.001** |
| EAT density (HU) | -87 (-92--83) | -88 (-92--84) | -87 (-91--83) | 0.43 |

AS, aortic stenosis; ACS, acute coronary syndrome; AVA, aortic valve area; BMI, body mass index; CAD, coronary artery disease; CKD, chronic kidney disease; EAT, epicardial adipose tissue; EF, ejection fraction; HG, high-gradient; HTN, hypertension; HU, Hounsfield units; LG, low-gradient; PG, pressure gradient; Unk: Unknown.

| **Supplemental Table 2. Echocardiographic characteristics that determine cardiac damage stage** | | | | | | | |
| --- | --- | --- | --- | --- | --- | --- | --- |
|  | **Total**  **(n=666)** | **Stage 0**  **(n=93)** | **Stage 1**  **(n=46)** | **Stage 2**  **(n=312)** | **Stage 3**  **(n=180)** | **Stage 4**  **(n=35)** | **p-value** |
| LV mass index (gr/m^2^) | 289 (46%) | 0 (0%) | 37 (84%) | 141(48%) | 93 (53%) | 18 (51%) | **<0.001** |
| Diastolic dysfunction grade II or III | 91(14%) | 0 (0%) | 5 (11%) | 41 (13%) | 37 (21%) | 8 (23%) | **<0.001** |
| EF (%) | 60 (55-65) | 65 (60-69) | 64 (52-65) | 60 (55-65) | 60 (55-65) | 45 (30-60) | **<0.001** |
| EF <50% | 108 (16%) | 0 (0%) | 7 (15%) | 53 (17%) | 28 (16%) | 20 (57%) | **<0.001** |
| Dilated LA | 467 (70%) | 0 (0%) | 0 (0%) | 284 (91%) | 152 (84%) | 31 (89%) | **<0.001** |
| Mod/Sev MR | 136 (20%) | 0 (0%) | 0 (0%) | 60 (19%) | 66 (37%) | 10 (29%) | **<0.001** |
| AF | 148 (22%) | 0 (0%) | 0 (0%) | 69 (22%) | 61 (34%) | 18 (51%) | **<0.001** |
| Severe PH | 26 (4%) | 0 (0%) | 0 (0%) | 0 (0%) | 20 (11%) | 6 (17%) | **<0.001** |
| Mod/Sev TR | 101 (15%) | 0 (0%) | 0 (0%) | 0 (0%) | 85 (47%) | 16 (46%) | **<0.001** |
| Mod/Severe RV  dysfunction | 35 (5%) | 0 (0%) | 0 (0%) | 0 (0%) | 0 (0%) | 35 (100%) | **<0.001** |

AF, atrial fibrilation; EF, ejection fraction; LA, left atrium; LV, left ventricular; MR, mitral regurgitation; Mod, moderate; PH, pulmonary hypertension; RV, right ventricular; Sev, severe; TR, tricuspid regurgitation.

| **Supplemental Table 3. Baseline characteristics of patients stratified by EAT volume** | | | | |
| --- | --- | --- | --- | --- |
|  | | | | |
|  | **Total** | **Low EAT volume**  **(<49 cm^3^)** | **High EAT volume**  **(≥49cm^3^)** | **p-value** |
|  | **N=666** | **N=229** | **N=437** |  |
| Age | 81 (74-86) | 82 (75-87) | 80 (74-85) | 0.055 |
| Sex |  |  |  | **<0.001** |
| Female | 357 (54%) | 154 (67%) | 203 (46%) |  |
| Race/ethnicity |  |  |  | **<0.001** |
| Non-Hispanic Black | 88 (13%) | 50 (22%) | 38 (9%) |  |
| Hispanic | 158 (24%) | 42 (18%) | 116 (27%) |  |
| Other/Unknown | 155 (23%) | 60 (26%) | 95 (22%) |  |
| Non-Hispanic White | 265 (40%) | 77 (34%) | 188 (43%) |  |
| BMI (kg/m^2^) | 28 (24-32) | 25 (23-29) | 29 (25-34) | **<0.001** |
| BMI Categories |  |  |  | **<0.001** |
| <25 kg/m^2^ | 213 (32%) | 108 (47%) | 105 (24%) |  |
| 25-30 kg/m^2^ | 230 (35%) | 75 (33%) | 155 (35%) |  |
| ≥30 kg/m^2^ | 223 (33%) | 46 (20%) | 177 (41%) |  |
| HTN | 320 (48%) | 117 (51%) | 203 (46%) | 0.26 |
| Diabetes | 151 (23%) | 47 (21%) | 104 (24%) | 0.34 |
| Dyslipidemia | 266 (40%) | 94 (41%) | 172 (39%) | 0.67 |
| CAD | 124 (19%) | 43 (19%) | 81 (19%) | 0.94 |
| ACS | 10 (2%) | 3 (1%) | 7 (2%) | 0.77 |
| CKD | 94 (14%) | 34 (15%) | 60 (14%) | 0.69 |
| Stroke | 37 (6%) | 13 (6%) | 24 (5%) | 0.92 |
| EF (%) | 60 (55-65) | 62 (55-65) | 60 (55-65) | 0.90 |
| AVA (continuity, cm^2^) | 0.73 (0.60-0.85) | 0.73 (0.59-0.84) | 0.73 (0.61-0.85) | 0.23 |
| Ao mean PG (mmHg) | 39 (31-46) | 39 (32-47) | 39 (31-46) | 0.73 |
| AS max vel (cm/s) | 400 (360-440) | 400 (361-442) | 400 (360-440) | 0.89 |
| Valve morphology |  |  |  | 0.18 |
| Bicuspid | 37 (6%) | 9 (4%) | 28 (6%) |  |
| AS Severity |  |  |  | 0.40 |
| LG-AS | 303 (45%) | 99 (43%) | 204 (47%) |  |
| HG-AS | 363 (55%) | 130 (57%) | 233 (53%) |  |
| Cardiac Damage Stage |  |  |  | **0.020** |
| 0 | 93 (14%) | 32 (14%) | 61 (14%) | 1.000 |
| 1 | 46 (7%) | 12 (5%) | 34 (8%) | 0.286 |
| 2 | 312 (47%) | 93 (41%) | 219 (50%) | **0.020** |
| 3 | 180 (27%) | 75 (33%) | 105 (24%) | **0.016** |
| 4 | 35 (5%) | 17 (7%) | 18 (4%) | 0.069 |
| EAT volume (cm^3^) | 63 (40-92) | 32 (25-40) | 81 (63-110) | **<0.001** |
| EAT density (HU) | -87 (-92--83) | -85 (-88--81) | -89 (-93--85) | **<0.001** |

AS, aortic stenosis; ACS, acute coronary syndrome; AVA, aortic valve area; BMI, body mass index; CAD, coronary artery disease; CKD, chronic kidney disease; EAT, epicardial adipose tissue; EF, ejection fraction; HG, high-gradient; HTN, hypertension; HU, Hounsfield units; LG, low-gradient; PG, pressure gradient.

| **Supplemental Table 4. Baseline characteristics according to AS hemodynamic type.** | | | | |
| --- | --- | --- | --- | --- |
|  | **Total**  **(n=666)** | **LG-AS**  **(n=303)** | **HG-AS**  **(n=363)** | **p-value** |
| Age | 81 (74-86) | 81 (75-86) | 80 (74-86) | 0.42 |
| Sex |  |  |  | **0.036** |
| Female | 357 (54%) | 149 (49%) | 208 (57%) |  |
| Race/ethnicity |  |  |  | 0.40 |
| Non-Hispanic Black | 88 (13%) | 41 (14%) | 47 (13%) |  |
| Hispanic | 158 (24%) | 80 (26%) | 78 (21%) |  |
| Other/Unknown | 155 (23%) | 64 (21%) | 91 (25%) |  |
| Non-Hispanic White | 265 (40%) | 118 (39%) | 147 (40%) |  |
| BMI (kg/m^2^) | 28 (24-32) | 27 (23-31) | 28 (24-33) | **0.007** |
| BMI Categories |  |  |  | **0.011** |
| <25 kg/m^2^ | 213 (32%) | 110 (36%) | 103 (28%) |  |
| 25-30 kg/m^2^ | 230 (35%) | 109 (36%) | 121 (33%) |  |
| ≥30 kg/m^2^ | 223 (33%) | 84 (28%) | 139 (38%) |  |
| HTN | 320 (48%) | 160 (53%) | 160 (44%) | **0.025** |
| Diabetes | 151 (23%) | 72 (24%) | 79 (22%) | 0.54 |
| Dyslipidemia | 266 (40%) | 132 (44%) | 134 (37%) | 0.081 |
| CAD | 124 (19%) | 73 (24%) | 51 (14%) | **<0.001** |
| ACS | 10 (2%) | 8 (3%) | 2 (1%) | **0.027** |
| CKD | 94 (14%) | 22 (7%) | 72 (20%) | **<0.001** |
| Stroke | 37 (6%) | 15 (5%) | 22 (6%) | 0.53 |
| EF (%) | 60 (55-65) | 60 (50-65) | 64 (57-65) | **0.002** |
| AVA (continuity, cm^2^) | 0.73 (0.60-0.85) | 0.78 (0.68-0.88) | 0.69 (0.56-0.80) | **<0.001** |
| Ao mean PG (mmHg) | 39 (31-46) | 30 (25-34) | 45 (41-53) | **<0.001** |
| AS max vel (cm/s) | 400 (360-440) | 357 (324-378) | 438 (409-464) | **<0.001** |
| Valve morphology |  |  |  | 0.95 |
| Bicuspid | 37 (6%) | 17 (6%) | 20 (6%) |  |
| Cardiac Damage Stages |  |  |  | **0.004** |
| 0 | 93 (14%) | 41 (14%) | 52 (14%) | 0.77 |
| 1 | 46 (7%) | 22 (7%) | 24 (7%) | 0.74 |
| 2 | 312 (47%) | 138 (46%) | 174 (48%) | 0.54 |
| 3 | 180 (27%) | 75 (25%) | 105 (29%) | 0.23 |
| 4 | 35 (5%) | 27 (9%) | 8 (2%) | **<0.001** |
| EAT volume (cm^3^) | 63 (40-92) | 62 (40-89) | 63 (40-94) | 0.87 |
| EAT volume ≥ 49cm^3^ | 437 (66%) | 204 (67%) | 233 (64%) | 0.40 |
| EAT density (HU) | -87 (-92--83) | -87 (-91--83) | -87 (-92--83) | 0.54 |

AS, aortic stenosis; ACS, acute coronary syndrome; AVA, aortic valve area; BMI, body mass index; CAD, coronary artery disease; CKD, chronic kidney disease; EAT, epicardial adipose tissue; EF, ejection fraction; HG, high-gradient; HTN, hypertension; HU, Hounsfield units; LG, low-flow low-gradient; PG, pressure gradient.

| **Supplemental Table 5. Baseline demographics per cardiac damage stages** | | | | | | | |
| --- | --- | --- | --- | --- | --- | --- | --- |
|  | **Total (n=666)** | **Stage 0 (n=93)** | **Stage 1 (n=46)** | **Stage 2 (n=312)** | **Stage 3 (n=180)** | **Stage 4 (n=35)** | **p-value** |
| Age | 81 (74-86) | 78 (71-83) | 80 (73-87) | 80 (74-86) | 82 (74-87) | 81 (74-85) | **0.037** |
| Sex |  |  |  |  |  |  | 0.38 |
| Female | 357 (54%) | 44 (47%) | 21 (46%) | 170 (54%) | 100 (56%) | 22 (63%) |  |
| Race/ethnicity |  |  |  |  |  |  | 0.29 |
| Non-Hispanic Black | 88 (13%) | 11 (12%) | 5 (11%) | 34 (11%) | 27 (15%) | 11 (31%) |  |
| Hispanic | 158 (24%) | 21 (23%) | 9 (20%) | 78 (25%) | 45 (25%) | 5 (14%) |  |
| Other/Unknown | 155 (23%) | 21 (23%) | 12 (26%) | 74 (24%) | 41 (23%) | 7 (20%) |  |
| Non-Hispanic White | 265 (40%) | 40 (43%) | 20 (43%) | 126 (40%) | 67 (37%) | 12 (34%) |  |
| BMI (kg/m^2^) | 28 (24-32) | 28 (24-31) | 28 (24-30) | 28 (24-32) | 28 (24-32) | 27 (23-32) | 0.94 |
| BMI Categories |  |  |  |  |  |  | 0.53 |
| <25 kg/m^2^ | 213 (32%) | 29 (31%) | 14 (30%) | 95 (30%) | 61 (34%) | 14 (40%) |  |
| 25-30 kg/m^2^ | 230 (35%) | 37 (40%) | 21 (46%) | 106 (34%) | 57 (32%) | 9 (26%) |  |
| ≥30 kg/m^2^ | 223 (33%) | 27 (29%) | 11 (24%) | 111 (36%) | 62 (34%) | 12 (34%) |  |
| HTN | 320 (48%) | 38 (41%) | 19 (41%) | 150 (48%) | 92 (51%) | 21 (60%) | 0.24 |
| Diabetes | 151 (23%) | 15 (16%) | 9 (20%) | 67 (21%) | 48 (27%) | 12 (34%) | 0.13 |
| Dyslipidemia | 266 (40%) | 33 (35%) | 12 (26%) | 130 (42%) | 75 (42%) | 16 (46%) | 0.24 |
| CAD | 124 (19%) | 18 (19%) | 8 (17%) | 61 (20%) | 35 (19%) | 2 (6%) | 0.38 |
| ACS | 10 (2%) | 1 (1%) | 1 (2%) | 4 (1%) | 3 (2%) | 1 (3%) | 0.94 |
| CKD | 94 (14%) | 9 (10%) | 2 (4%) | 41 (13%) | 36 (20%) | 6 (17%) | **0.029** |
| Stroke | 37 (6%) | 4 (4%) | 3 (7%) | 19 (6%) | 10 (6%) | 1 (3%) | 0.91 |
| EF (%) | 60 (55-65) | 65 (60-69) | 64 (52-65) | 60 (55-65) | 60 (55-65) | 45 (30-60) | **<0.001** |
| AVA (continuity, cm^2^) | 0.73 (0.60-0.85) | 0.75 (0.65-0.88) | 0.73 (0.66-0.83) | 0.72 (0.60-0.85) | 0.73 (0.60-0.86) | 0.68 (0.58-0.80) | 0.27 |
| Ao mean PG (mmHg) | 39 (31-46) | 38 (33-45) | 40 (34-47) | 40 (31-47) | 40 (32-48) | 30 (23-37) | **<0.001** |
| AS max vel (cm/s) | 400 (360-440) | 400 (370-430) | 380 (356-427) | 402 (360-445) | 400 (360-452) | 378 (321-434) | 0.48 |
| Valve morphology |  |  |  |  |  |  | 0.80 |
| Bicuspid | 37 (6%) | 6 (6%) | 2 (4%) | 20 (6%) | 7 (4%) | 2 (6%) |  |
| AS type |  |  |  |  |  |  | **0.004** |
| LG-AS | 303 (45%) | 41 (44%) | 22 (48%) | 138 (44%) | 75 (42%) | 27 (77%) |  |
| HG-AS | 363 (55%) | 52 (56%) | 24 (52%) | 174 (56%) | 105 (58%) | 8 (23%) |  |

AS, aortic stenosis; ACS, acute coronary syndrome; AVA, aortic valve area; BMI, body mass index; CAD, coronary artery disease; CKD, chronic kidney disease; EAT, epicardial adipose tissue; EF, ejection fraction; HG, high-gradient; HTN, hypertension; HU, Hounsfield units; LG, low-flow low-gradient; PG, pressure gradient.

| **Supplemental Table 6. Baseline characteristics of patients stratified by EAT density** | | | | |
| --- | --- | --- | --- | --- |
|  | **Total** | **EAT density**  **(<-86HU)** | **EAT density**  **(≥-86HU)** | **p-value** |
|  | **N=666** | **N=380** | **N=286** |  |
| Age | 81 (74-86) | 79 (74-84) | 82 (75-87) | **0.002** |
| Sex |  |  |  | 0.60 |
| Female | 357 (54%) | 207 (54%) | 150 (52%) |  |
| Race/Ethnicity |  |  |  | 0.57 |
| Non-Hispanic Black | 88 (13%) | 46 (12%) | 42 (15%) |  |
| Hispanic | 158 (24%) | 89 (23%) | 69 (24%) |  |
| Other/Unknown | 155 (23%) | 95 (25%) | 60 (21%) |  |
| Non-Hispanic White | 265 (40%) | 150 (39%) | 115 (40%) |  |
| BMI (kg/m^2^) | 28 (24-32) | 29 (26-34) | 25 (23-29) | **<0.001** |
| BMI categories |  |  |  | **<0.001** |
| 0 (<25 kg/m^2^) | 213 (32%) | 79 (21%) | 134 (47%) |  |
| 1 (25-30 kg/m^2^) | 230 (35%) | 127 (33%) | 103 (36%) |  |
| 2 (≥30 kg/m^2^) | 223 (33%) | 174 (46%) | 49 (17%) |  |
| HTN | 320 (48%) | 182 (48%) | 138 (48%) | 0.93 |
| Diabetes | 151 (23%) | 92 (24%) | 59 (21%) | 0.27 |
| Dyslipidemia | 266 (40%) | 151 (40%) | 115 (40%) | 0.90 |
| CAD | 124 (19%) | 76 (20%) | 48 (17%) | 0.29 |
| ACS | 10 (2%) | 7 (2%) | 3 (1%) | 0.40 |
| CKD | 94 (14%) | 43 (11%) | 51 (18%) | **0.017** |
| Stroke | 37 (6%) | 15 (4%) | 22 (8%) | **0.037** |
| EF (%) | 60 (55-65) | 62 (55-65) | 60 (55-65) | 0.40 |
| AVA (continuity, cm^2^) | 0.73 (0.60-0.85) | 0.73 (0.63-0.85) | 0.72 (0.57-0.84) | 0.055 |
| Ao mean PG (mmHg) | 39 (31-46) | 39 (32-47) | 39 (31-46) | 0.63 |
| AS max vel (cm/s) | 400 (360-440) | 400 (360-440) | 400 (370-442) | 0.82 |
| Valve morphology |  |  |  | 0.47 |
| Bicuspid | 37 (6%) | 19 (5%) | 18 (6%) |  |
| AS severity |  |  |  | 0.77 |
| LG-AS | 303 (45%) | 171 (45%) | 132 (46%) |  |
| HG-AS | 363 (55%) | 209 (55%) | 154 (54%) |  |
| Cardiac Damage Stage |  |  |  | **<0.001** |
| 0 | 93 (14%) | 67 (18%) | 26 (9%) | **0.002** |
| 1 | 46 (7%) | 24 (6%) | 22 (8%) | 0.49 |
| 2 | 312 (47%) | 198 (52%) | 114 (40%) | **0.002** |
| 3 | 180 (27%) | 77 (20%) | 103 (36%) | **<0.001** |
| 4 | 35 (5%) | 14 (4%) | 21 (7%) | **0.036** |

AS, aortic stenosis; ACS, acute coronary syndrome; AVA, aortic valve area; BMI, body mass index; CAD, coronary artery disease; CKD, chronic kidney disease; EAT, epicardial adipose tissue; EF, ejection fraction; HG, high-gradient; HTN, hypertension; HU, Hounsfield units; LG, low-flow low-gradient; PG, pressure gradient.


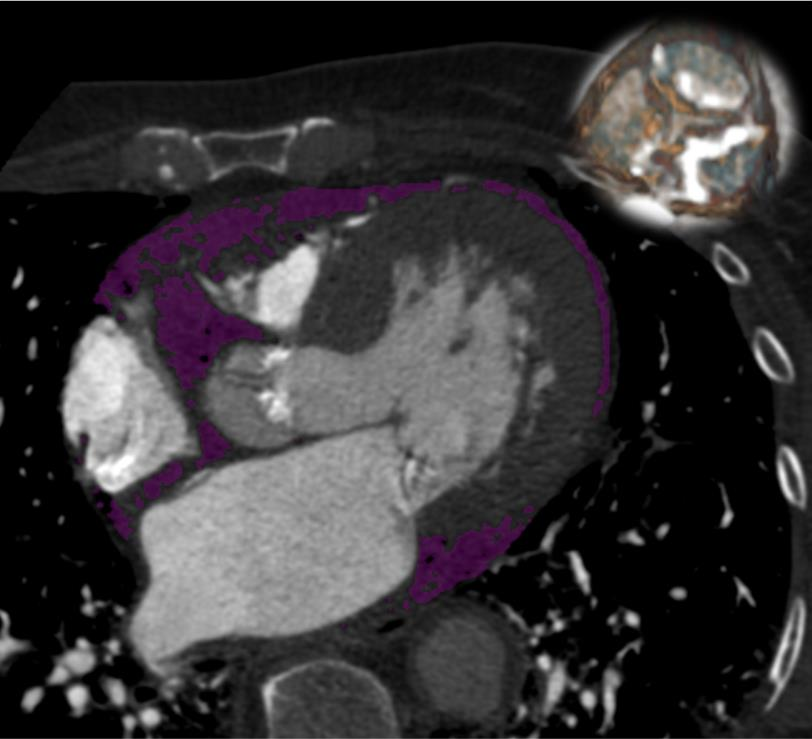


**Supplemental Figure 1.** Deep-learning-based EAT segmentation example in a patient with severe calcific AS.


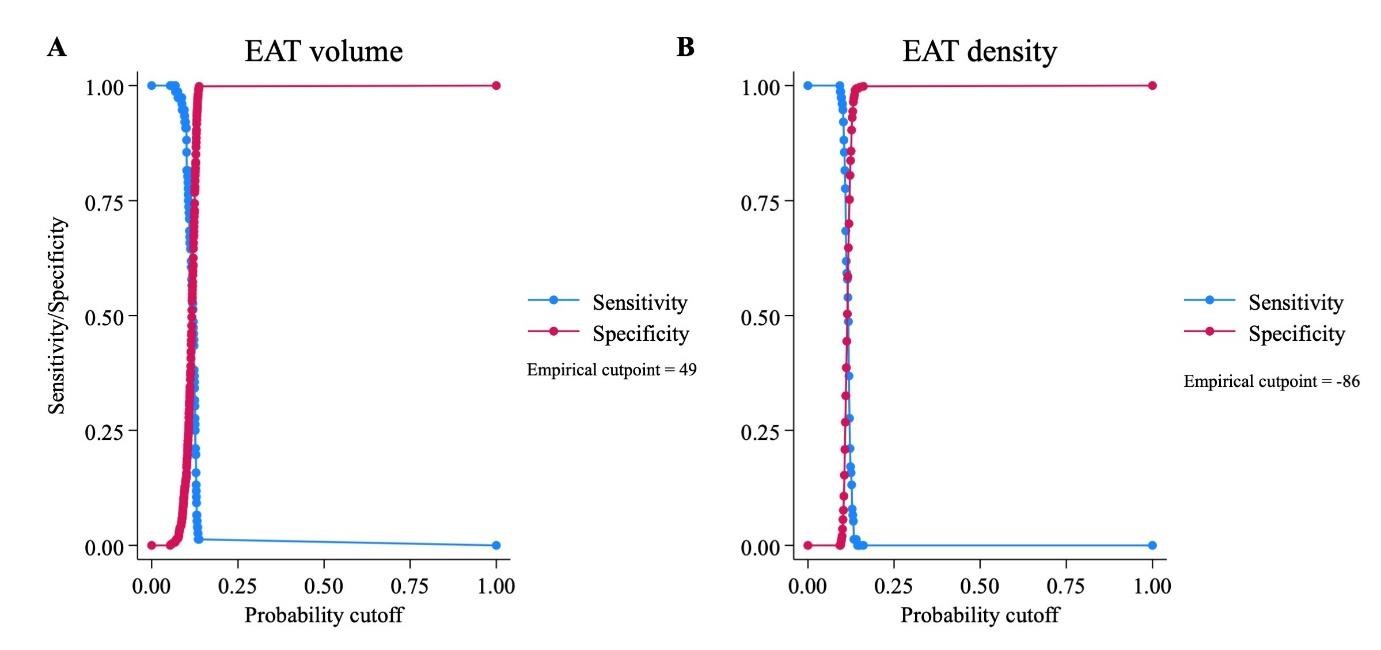


**Supplemental Figure 2.** Cutoff estimation using the Youden Index for (A) epicardial adipose tissue (EAT) volume and (B) EAT density.


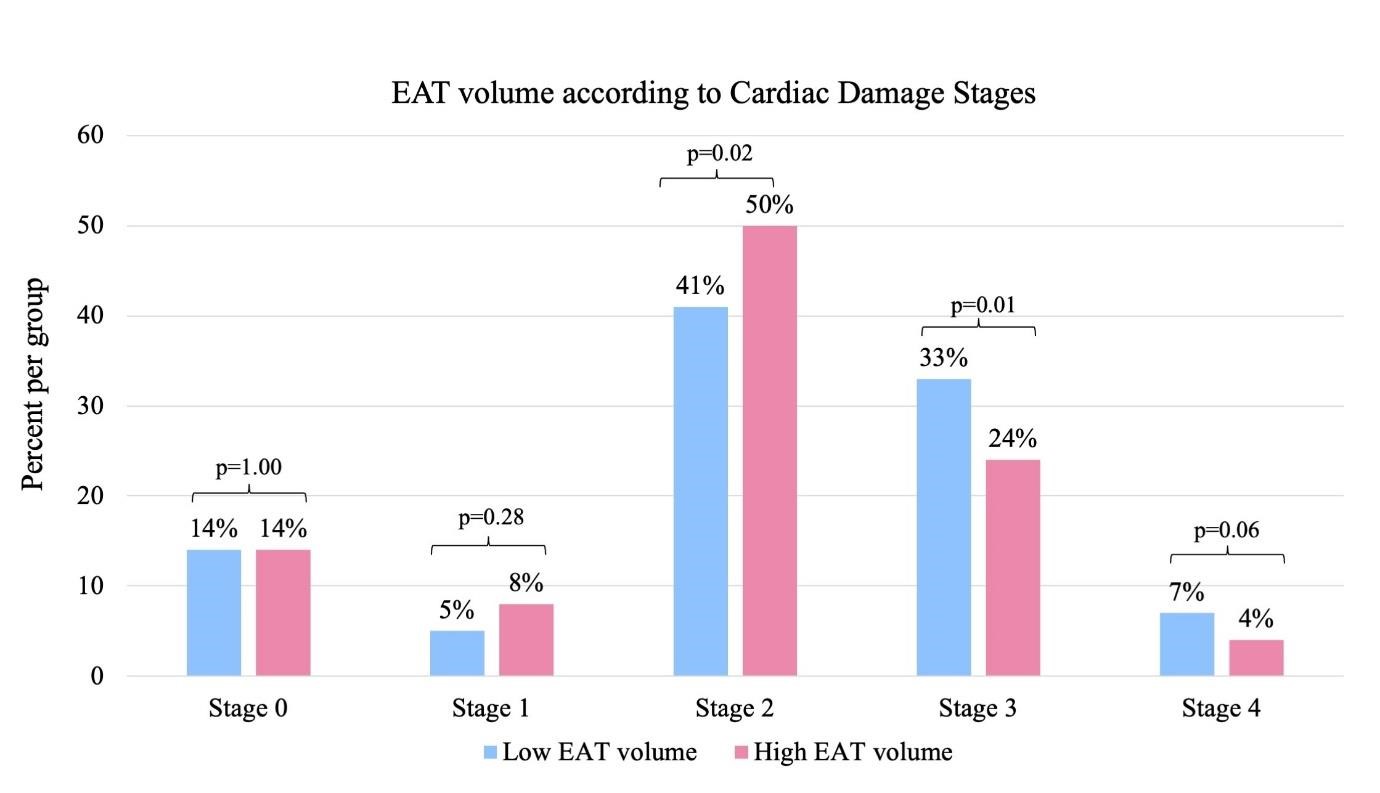


**Supplemental Figure 3.** EAT volume distribution according to cardiac damage stages. EAT, epicardial adipose tissue.


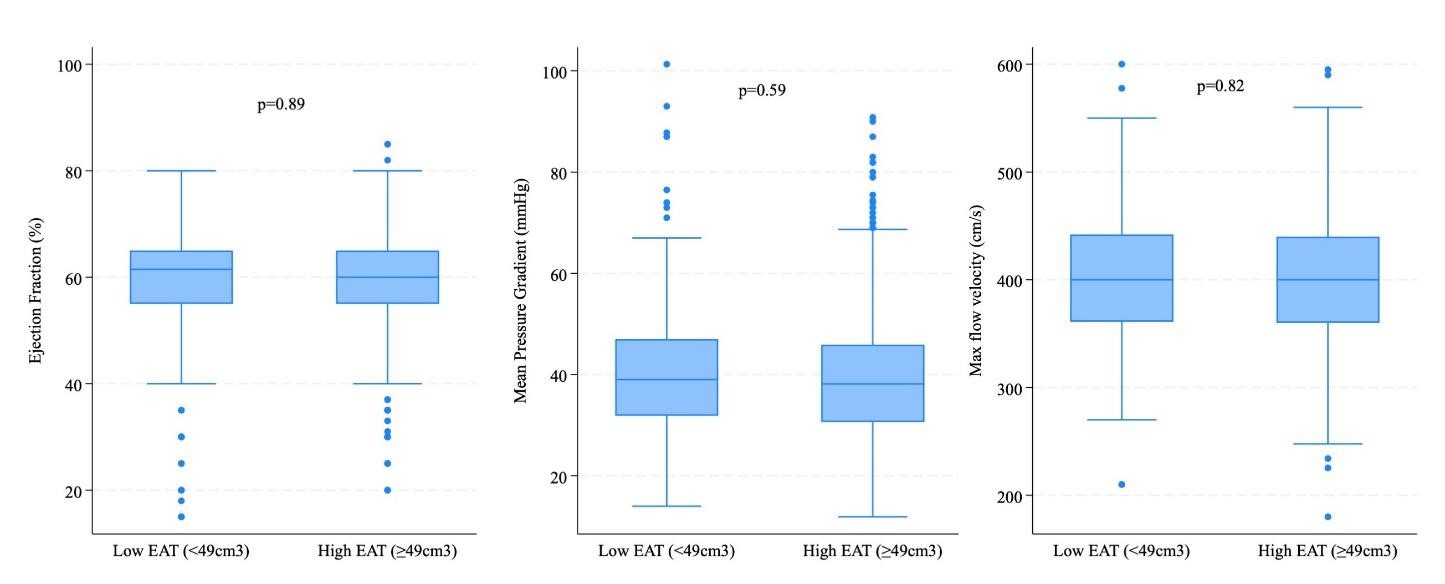


**Supplemental Figure 4.** Baseline echocardiographic parameters in patients with high and low EAT volumes. The image shows the ejection fraction median (interquartile range), mean aortic pressure gradient, and maximum aortic flow velocity, with no differences between the two groups.


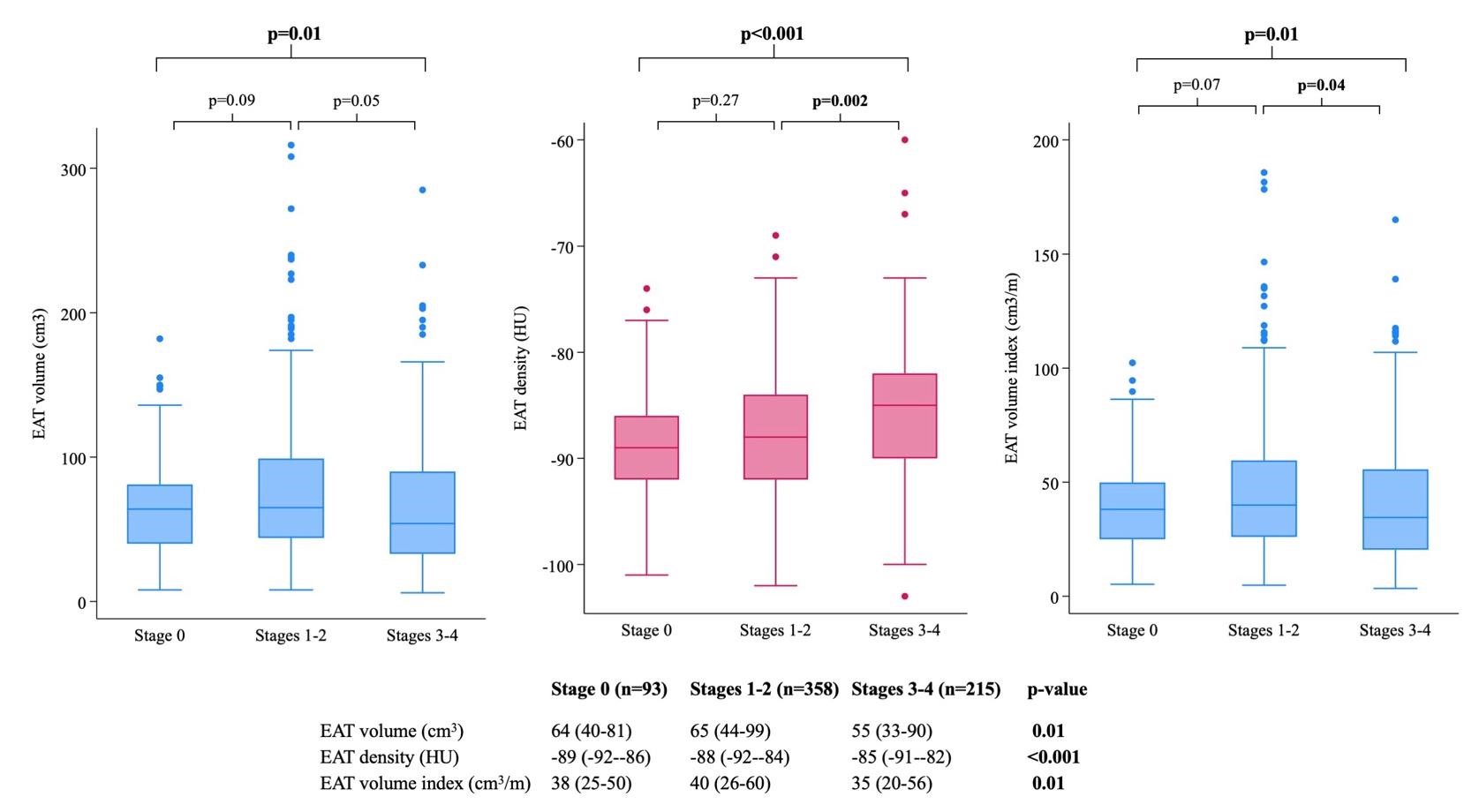


**Supplemental Figure 5.** Epicardial adipose tissue volume, density, and volume index by height according to cardiac damage stages. EAT, epicardial adipose tissue

**
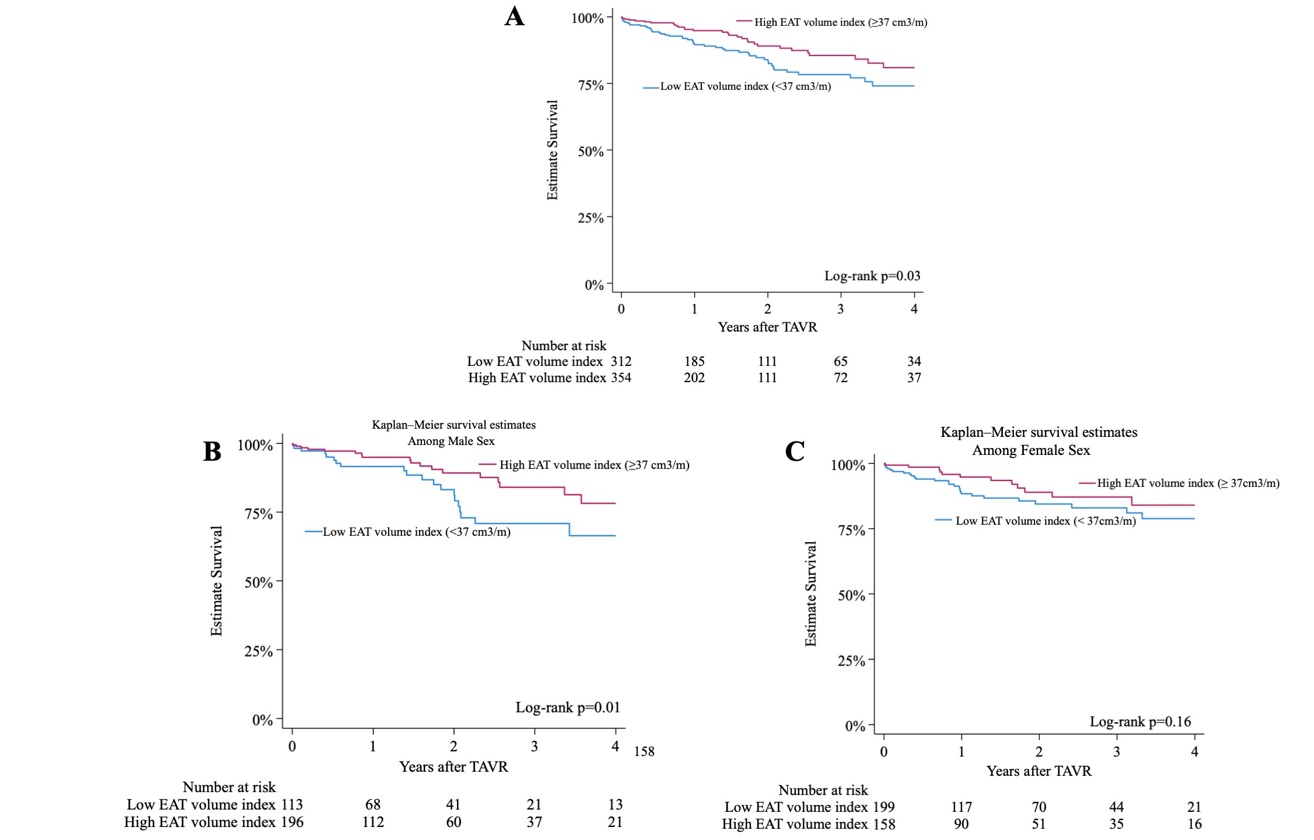
**

**Supplemental Figure 6.** **Kaplan-Meier graph demonstrating four-year survival for EAT volume index with (A) estimated cutoff for the overall cohort and (B-C) stratified by sex.** EAT, epicardial adipose tissue.


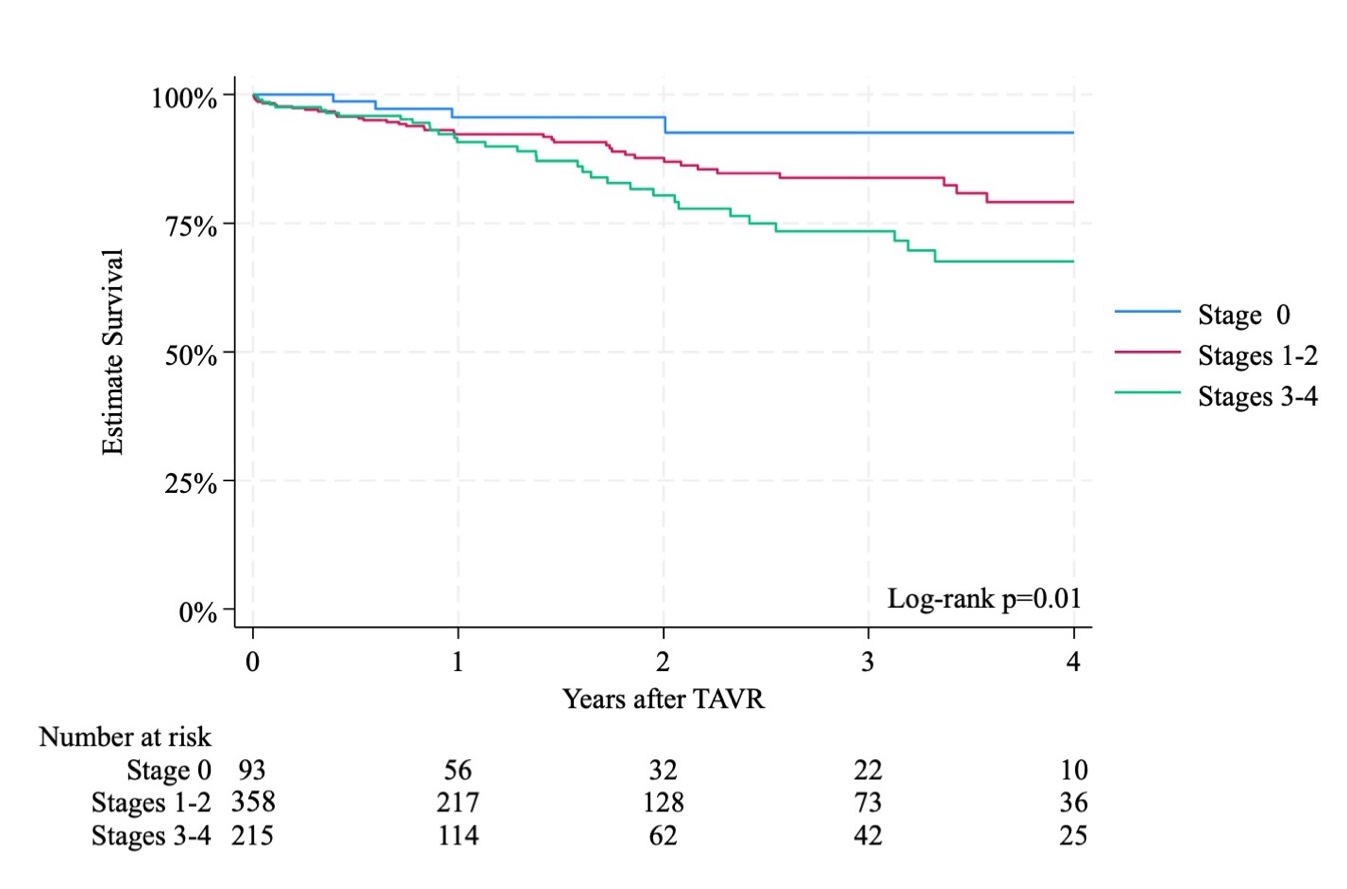


**Supplemental Figure 7.** **Kaplan-Meier graph demonstrating four-year survival according to cardiac damage stage.**


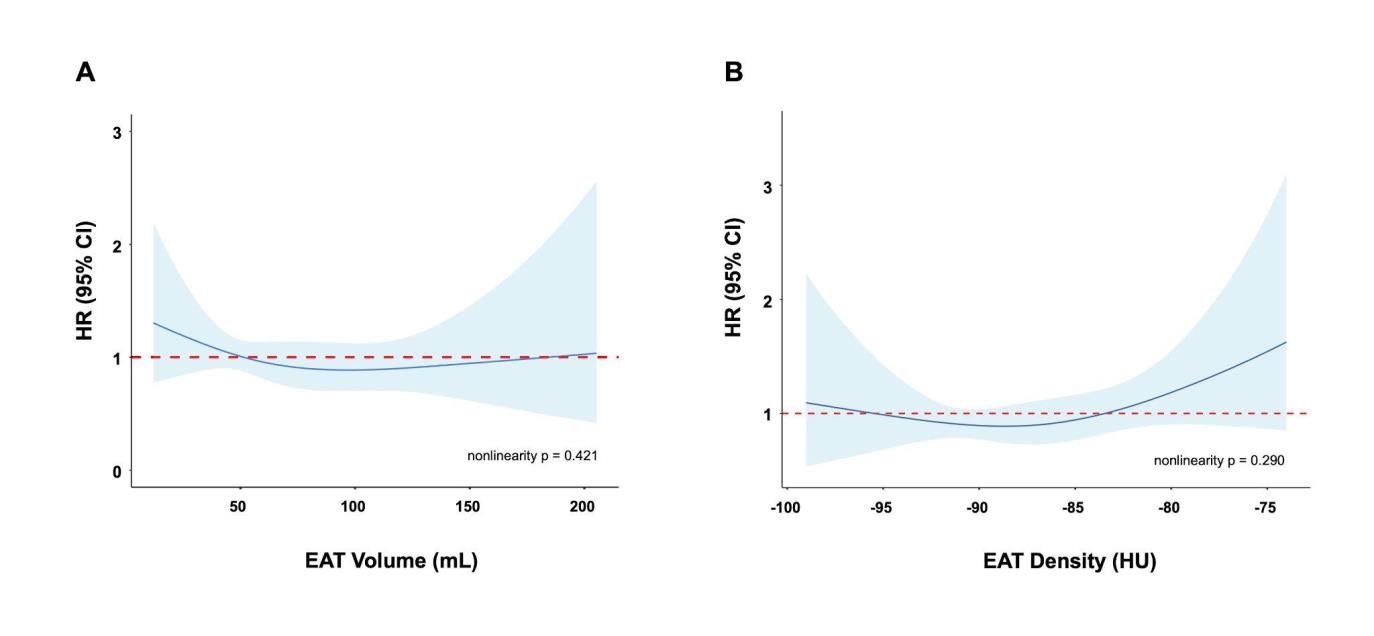


**Supplemental Figure 8**. **Association between Epicardial Adipose Tissue Volume (A) and density (B) and mortality**. CI, confidence interval; EAT, epicardial adipose tissue; HR, hazard ratio; HU, Hounsfield Units.
